# Supplementary material for: HIVprotI: an integrated web based platform for prediction and design of HIV proteins inhibitors
Source: J Cheminform. 2018 Mar 9;10:12. doi: 10.1186/s13321-018-0266-y (PMC5845081; doi:10.1186/s13321-018-0266-y)
Supplement: Supplementary file 2 — Additional file 2. Source code of HIVProtI web server. [file 13321_2018_266_MOESM2_ESM.zip › HIVprotI_Source-code/hivprotI/weka-3-6-6/doc/weka/classifiers/functions/supportVector/PolyKernel.html]

PolyKernel


---


|  |  |  |
| --- | --- | --- |
| **Overview** \| **Package** \| **Class** \| **Tree** \| **Deprecated** \| **Index** \| **Help** \| **Weka's home** | |  |
| **PREV CLASS**   **NEXT CLASS** | **FRAMES**    **NO FRAMES**     **All Classes** |  |
| SUMMARY: NESTED \| FIELD \| CONSTR \| METHOD | DETAIL: FIELD \| CONSTR \| METHOD |  |


---


## weka.classifiers.functions.supportVector Class PolyKernel

```
java.lang.Object
  weka.classifiers.functions.supportVector.Kernel
      weka.classifiers.functions.supportVector.CachedKernel
          weka.classifiers.functions.supportVector.PolyKernel
```

**All Implemented Interfaces:**: java.io.Serializable, CapabilitiesHandler, OptionHandler, RevisionHandler

**Direct Known Subclasses:**: MIPolyKernel, NormalizedPolyKernel

---

``` public class PolyKernel extends CachedKernel ```

The polynomial kernel : K(x, y) = <x, y>^p or K(x, y) = (<x, y>+1)^p


Valid options are:

```
 -D
  Enables debugging output (if available) to be printed.
  (default: off)
```

```
 -no-checks
  Turns off all checks - use with caution!
  (default: checks on)
```

```
 -C <num>
  The size of the cache (a prime number), 0 for full cache and 
  -1 to turn it off.
  (default: 250007)
```

```
 -E <num>
  The Exponent to use.
  (default: 1.0)
```

```
 -L
  Use lower-order terms.
  (default: no)
```

**Version:**
:   $Revision: 5808 $

**Author:**
:   Eibe Frank (eibe@cs.waikato.ac.nz), Shane Legg (shane@intelligenesis.net) (sparse vector code), Stuart Inglis (stuart@reeltwo.com) (sparse vector code)

**See Also:**: Serialized Form

---

**Constructor Summary**

`PolyKernel()`
  
          default constructor - does nothing.

`PolyKernel(Instances data,
int cacheSize,
double exponent,
boolean lowerOrder)`
  
          Creates a new `PolyKernel` instance.


| **Method Summary** | |
| --- | --- |
| `java.lang.String` | `exponentTipText()`             Returns the tip text for this property |
| `Capabilities` | `getCapabilities()`             Returns the Capabilities of this kernel. |
| `double` | `getExponent()`             Gets the exponent value. |
| `java.lang.String[]` | `getOptions()`             Gets the current settings of the Kernel. |
| `java.lang.String` | `getRevision()`             Returns the revision string. |
| `boolean` | `getUseLowerOrder()`             Gets whether lower-order terms are used. |
| `java.lang.String` | `globalInfo()`             Returns a string describing the kernel |
| `java.util.Enumeration` | `listOptions()`             Returns an enumeration describing the available options. |
| `void` | `setExponent(double value)`             Sets the exponent value. |
| `void` | `setOptions(java.lang.String[] options)`             Parses a given list of options. |
| `void` | `setUseLowerOrder(boolean value)`             Sets whether to use lower-order terms. |
| `java.lang.String` | `toString()`             returns a string representation for the Kernel |
| `java.lang.String` | `useLowerOrderTipText()`             Returns the tip text for this property |

**Methods inherited from class weka.classifiers.functions.supportVector.CachedKernel**

`buildKernel, cacheSizeTipText, clean, eval, getCacheSize, numCacheHits, numEvals, setCacheSize`

**Methods inherited from class weka.classifiers.functions.supportVector.Kernel**

`checksTurnedOffTipText, debugTipText, forName, getChecksTurnedOff, getDebug, makeCopies, makeCopy, setChecksTurnedOff, setDebug`

**Methods inherited from class java.lang.Object**

`equals, getClass, hashCode, notify, notifyAll, wait, wait, wait`

**Constructor Detail**

### PolyKernel

```
public PolyKernel()
```

:   default constructor - does nothing.

---


### PolyKernel

```
public PolyKernel(Instances data,
                  int cacheSize,
                  double exponent,
                  boolean lowerOrder)
           throws java.lang.Exception
```

:   Creates a new `PolyKernel` instance.

    **Parameters:**: `data` - the training dataset used.: `cacheSize` - the size of the cache (a prime number): `exponent` - the exponent to use: `lowerOrder` - whether to use lower-order terms **Throws:**: `java.lang.Exception` - if something goes wrong


**Method Detail**

### globalInfo

```
public java.lang.String globalInfo()
```

:   Returns a string describing the kernel

    :   **Specified by:**: `globalInfo` in class `Kernel`
    :   **Returns:**: a description suitable for displaying in the explorer/experimenter gui

---


### listOptions

```
public java.util.Enumeration listOptions()
```

:   Returns an enumeration describing the available options.

    :   **Specified by:**: `listOptions` in interface `OptionHandler` **Overrides:**: `listOptions` in class `CachedKernel`
    :   **Returns:**: an enumeration of all the available options.

---


### setOptions

```
public void setOptions(java.lang.String[] options)
                throws java.lang.Exception
```

:   Parses a given list of options.


    Valid options are:

    ```
     -D
      Enables debugging output (if available) to be printed.
      (default: off)
    ```

    ```
     -no-checks
      Turns off all checks - use with caution!
      (default: checks on)
    ```

    ```
     -C <num>
      The size of the cache (a prime number), 0 for full cache and 
      -1 to turn it off.
      (default: 250007)
    ```

    ```
     -E <num>
      The Exponent to use.
      (default: 1.0)
    ```

    ```
     -L
      Use lower-order terms.
      (default: no)
    ```

    :   **Specified by:**: `setOptions` in interface `OptionHandler` **Overrides:**: `setOptions` in class `CachedKernel`
    :   **Parameters:**: `options` - the list of options as an array of strings **Throws:**: `java.lang.Exception` - if an option is not supported

---


### getOptions

```
public java.lang.String[] getOptions()
```

:   Gets the current settings of the Kernel.

    :   **Specified by:**: `getOptions` in interface `OptionHandler` **Overrides:**: `getOptions` in class `CachedKernel`
    :   **Returns:**: an array of strings suitable for passing to setOptions

---


### getCapabilities

```
public Capabilities getCapabilities()
```

:   Returns the Capabilities of this kernel.

    :   **Specified by:**: `getCapabilities` in interface `CapabilitiesHandler` **Overrides:**: `getCapabilities` in class `Kernel`
    :   **Returns:**: the capabilities of this object **See Also:**: `Capabilities`

---


### setExponent

```
public void setExponent(double value)
```

:   Sets the exponent value.

    :   **Parameters:**: `value` - the exponent value

---


### getExponent

```
public double getExponent()
```

:   Gets the exponent value.

    :   **Returns:**: the exponent value

---


### exponentTipText

```
public java.lang.String exponentTipText()
```

:   Returns the tip text for this property

    :   **Returns:**: tip text for this property suitable for displaying in the explorer/experimenter gui

---


### setUseLowerOrder

```
public void setUseLowerOrder(boolean value)
```

:   Sets whether to use lower-order terms.

    :   **Parameters:**: `value` - true if lower-order terms will be used

---


### getUseLowerOrder

```
public boolean getUseLowerOrder()
```

:   Gets whether lower-order terms are used.

    :   **Returns:**: true if lower-order terms are used

---


### useLowerOrderTipText

```
public java.lang.String useLowerOrderTipText()
```

:   Returns the tip text for this property

    :   **Returns:**: tip text for this property suitable for displaying in the explorer/experimenter gui

---


### toString

```
public java.lang.String toString()
```

:   returns a string representation for the Kernel

    :   **Overrides:**: `toString` in class `java.lang.Object`
    :   **Returns:**: a string representaiton of the kernel

---


### getRevision

```
public java.lang.String getRevision()
```

:   Returns the revision string.

    :   **Specified by:**: `getRevision` in interface `RevisionHandler` **Overrides:**: `getRevision` in class `Kernel`
    :   **Returns:**: the revision


---


|  |  |  |
| --- | --- | --- |
| **Overview** \| **Package** \| **Class** \| **Tree** \| **Deprecated** \| **Index** \| **Help** \| **Weka's home** | |  |
| **PREV CLASS**   **NEXT CLASS** | **FRAMES**    **NO FRAMES**     **All Classes** |  |
| SUMMARY: NESTED \| FIELD \| CONSTR \| METHOD | DETAIL: FIELD \| CONSTR \| METHOD |  |


---
